# Spontaneous subdural hematoma associated with preeclampsia: a case report and litterature review

**DOI:** 10.11604/pamj.2014.19.213.5451

**Published:** 2014-10-28

**Authors:** Nezha Oudghiri, Mehdi Behat, Nada Elchhab, Mouhssine Doumiri, Anas Saoud Tazi

**Affiliations:** 1 Department of Obstetrics Intensive Care Unit and Anesthesia, Maternity Souissi Hospital, University Mohamed V, Rabat, Morocco

**Keywords:** spontaneous subdural hematoma, pre-eclampsia, neurologic

## Abstract

A patient with pre-eclampsia at 31 weeks’ gestation developed neurologic signs. Computerized tomography revealed a large cranial subdural hematoma. This diagnostic should be considered in any pre-eclamptic patient demonstrating neurological symptoms and must be treated effectively because of the poor maternel and fetal prognosis. Our patient was succesfully treated.

## Introduction

Intracranial hemorrhage is a rare complication during pregnancy, but potentially fatal, which contributes significantly to maternal mortality. The main causes are ruptured aneurysm, arteriovenous malformations's (MAV) and pregnancy-induced hypertension. The aneurysm or AVM usually causes a subarachnoid hemorrhage; intracranial hemorrhage while associated with pre-eclampsia is usually intra- parenchymal. Subdural hemorrhage associated with pregnancy has been reported in post trauma or as a complication of epidural anesthesia during labor. A spontaneous subdural hematoma associated with preeclampsia, have been reported in some cases in the literature. We now describe such a case.

## Patient and observation

**Figure 1:**
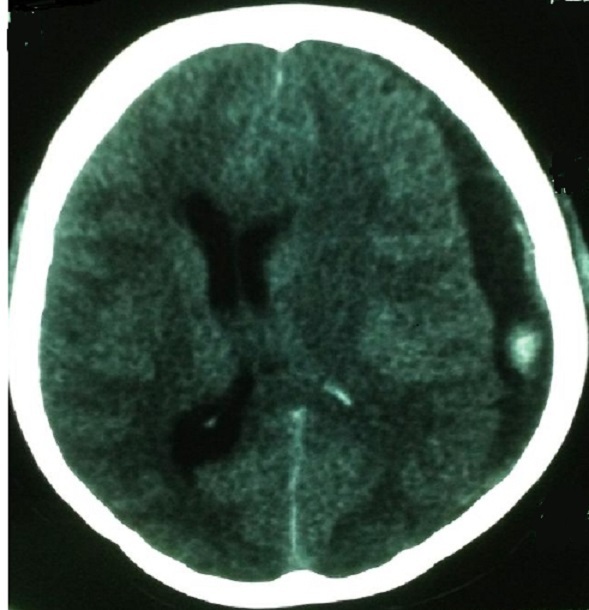
Computerized tomography scan of the head demonstrating a midline shift and a large left sided subdural hematoma

A 30-year old primigravid woman with no particular history was hospitalized for management of preeclampsia in pregnancy at 31weeks’ gestation. On admission the patient was sleepy with the the glascow coma scale at 13, eye examination revealed pupils to be equal and reactive to light bilaterally, blood pressure was 150/100 mmHg with 4+proteinuria without signs of trauma, no concept of convulsions, or tongue biting, the patient denied any headache, visual disturbances or epigastric pain. The cardiopulmonary examination was unremarkable. The obstetric examination note long neck and posterior closed and intact membrane. Obstetric ultrasound reveale an estimated fetal age of 27 weeks’ with adequate fluid volume. Laboratory studies included a hemoglobin of 13,6g/l, platelet count 178000/mm^3^, serum glutamic oxaloacetic transaminanse (SGOT): 11 IU/L, glutamic puryvate transaminase (SGPT): 15IU/L, urea: 0, 4 g/l, créatinine: 6.6mg/dl, total bilirubin: 8mg/dl. The brain scan objective a subdural hematoma (Figure 1). A first dose of betamethasone was administrated to enhance fetal lung maturity. The patient was admitted on the same day in the operating room and the hematoma was evacuated. The evolution was marked by neurological improvement without any motor or sensory deficit; she was well oriented and responsive to questions and commands. At the fifth day the parturient has delivered naturelly a 32 weeks’ newborn who died in the ICU because of respiratory distress and prematurity.

## Discussion

Intracranial hemorrhage is a rare complication occuring in 0.01-0.05 per cent in pregnancies. The aneurysm or arterio-venous malformations are the most common causes of intracranial hemorrhage. Pregnancy-induced hypertension is also a factor of intra parenchymal hemorrhage [1]. The subdural hematoma is a rare form of intracranial hemorrhage associated with pregnancy. Some cases subdural hematoma resulting from a head injury during pregnancy has been reported [2]. Other cases of subdural hematoma have been reported in post- partum in association with epidural anesthesia [3].

The clinical symptoms described in these patients in post partum presented by: headache, dizziness, disorientation, memory loss, ophthalmoplegia, papilledema, stupor, coma, and psychosis. The onset of these symptoms varies from the first to the fourth day after delivery [3]. It seems clear that it exists an association between trauma, whether related to a direct head injury or secondary to regional anesthesia and the development of a subdural hematoma. An association between pre-eclampsia and spontaneous subdural hematoma has not been previously reported. Gregg Giannina and al described a subdural hematoma during pregnancy in a patient with preeclampsia without notion of trauma. The exact etiology of the hematoma in this patient is not clear. However, thrombocytopenia may have been a predisposing factor for the development of intracranial hemorrhage and / or the inhibition of platelet function due to magnesium sulfate [4]. In our case the patient had a normal count of platelet and did not receive magnesium sulfate before.

## Conclusion

The hemorrhagic cerebrovascular accidents during pregnancy are rare, possibly involving maternal and fetal prognosis. They must be detected early and treated effectively, and often requires a multidisciplinary approach.
